# MicroRNAs in Inflammatory Heart Diseases and Sepsis-Induced Cardiac Dysfunction: A Potential Scope for the Future?

**DOI:** 10.3390/cells8111352

**Published:** 2019-10-30

**Authors:** Moritz Mirna, Vera Paar, Richard Rezar, Albert Topf, Miriam Eber, Uta C. Hoppe, Michael Lichtenauer, Christian Jung

**Affiliations:** 1 Clinic of Internal Medicine II, Department of Cardiology, Paracelsus Medical University of Salzburg, 5020 Salzburg, Austria; v.paar@salk.at (V.P.); r.rezar@salk.at (R.R.); m.eber@salk.at (M.E.); m.lichtenauer@salk.at (M.L.); 2 Division of Cardiology, Pulmonology, and Vascular Medicine, Medical Faculty, University Duesseldorf, 40225 Duesseldorf, Germany; christian.jung@med.uni-duesseldorf.de

**Keywords:** microRNA, miRNA, inflammatory heart diseases, sepsis-induced cardiac dysfunction, myocarditis, pericarditis, endocarditis

## Abstract

Background: MicroRNAs (miRNAs) are small, single-stranded RNA sequences that regulate gene expression on a post-transcriptional level. In the last few decades, various trials have investigated the diagnostic and therapeutic potential of miRNAs in several disease entities. Here, we provide a review of the available evidence on miRNAs in inflammatory heart diseases (myocarditis, endocarditis, and pericarditis) and sepsis-induced cardiac dysfunction. Methods: Systematic database research using the PubMed and Medline databases was conducted between July and September 2019 using predefined search terms. The whole review was conducted based on the Preferred Reporting Items for Systematic Reviews and Meta-Analyses (PRISMA) guidelines. Results: In total, 131 studies were screened, 96 abstracts were read, and 69 studies were included in the review. Discussion: In the future, circulating miRNAs could serve as biomarkers for diagnosis and disease monitoring in the context of inflammatory heart diseases and sepsis-induced cardiac dysfunction. Considering the promising results of different animal models, certain miRNAs could also emerge as novel therapeutic approaches in this setting.

## 1. Introduction

The term “microRNAs” (miRNAs) summarizes a class of small ribonucleic acids (RNAs) (19–24 nucleotides) characterized by a distinctive intramolecular “hairpin-loop” structure that have been found in various eukaryotic organisms. Deriving from primary miRNAs (pri-miRNAs), they undergo processing via the two well-known RNAse III enzyme complexes “Drosha” and “Dicer”. Later, the mature miRNAs bind to RNA-induced silencing complexes (RISC) and lead them to complementary regions in messenger RNAs (mRNAs). Although classified as “noncoding”, miRNAs play an important role in post-transcriptional gene silencing (PTGS) and, consequently, protein synthesis or omittance. Interestingly, certain miRNAs are highly expressed in healthy cardiac tissue and thus probably play a role in the maintenance of normal cardiac function (miR-1, miR-16, miR-27b, miR-30d, miR-126, miR-133, miR-143, and others) [1], while others have been associated with inflammatory and cardiovascular diseases [2,3,4]. Targeting tens to hundreds of transcripts each, the role of different miRNAs in a variety of physiological and pathophysiological pathways and, nowadays, their use as biomarkers, as well as therapeutic targets, are the subject of extensive research [1,5,6].

### MicroRNAs and Their Role in Inflammatory Conditions

Current evidence suggests that the detection of different pathogen- or microbe-associated molecular patterns (PAMPs/MAMPs), such as lipopolysaccharides (LPS), nucleic acids, and/or proteins by so-called pattern-recognition receptors (PPRs), enables adequate and timely response to pathogens by the innate immune system. Among the PPRs, several investigative trials in the field of cellular immunology have addressed the family of toll-like receptors (TLRs). Different cellular and humoral pro- and anti-inflammatory mediators are involved in providing a balanced inflammatory response to injury and infection. The interaction of TLRs with miRNAs, as well as their role in the regulation and fine-tuning of certain immunological pathways, has been investigated in the last decade. To elucidate the complex role of miRNAs in inflammatory processes in general, a model is provided below to explain the interactions of the individual players involved.

One important mechanism in inflammatory and autoimmune processes is the TLR4-/MYD88-pathway, as it induces nuclear factor kappa B (NFκB)-dependent gene transcription, thus initiating the inflammatory cascade. Notably, TLR4 signaling also increases the expression of different miRNAs, such as miR-155 or miR-21, which results in an increase in interleukin (IL)-10 levels and, consequently, in a downregulation of TLR4-mediated signaling. Interestingly, miR-155 on the one hand provides stabilizing effects on tumor necrosis factor (TNF) mRNA, whereas, on the other hand, its overexpression was found to negatively influence the transcription of IL-1-dependent genes by targeting TAK1-binding protein 2 (TAB2) [7]. Recently, Curtale et al. identified the IL-10/transforming growth factor (TGF)-β-induced cluster of two miRNAs—miR-125a-5p and let-7e-5p—as another key element in the downregulation of inflammatory responses, hence contributing to an adequate host response. The so-called “miR-125a~99b~let-7e” cluster was shown to be negatively influenced by interferon (IFN)-γ [8], which itself represents an important activator of the cellular immune response [9]. Thus, not only processes are initiated and regulated by negative feedback loops via the expression and interaction of these small molecules but also pro- and anti-inflammatory pathways are simultaneously activated. Consequently, an inflammatory response is not a "suicide mission" according to the "all or nothing" principle but a complex balance between host defense and tolerance. In the following sections, the specific immunological roles of miRNAs, with respect to inflammatory heart diseases and sepsis-induced cardiac dysfunction, are discussed.

## 2. Materials and Methods

Systematic database research using the PubMed and Medline databases was conducted between July and September 2019. The search terms used are listed in Appendix A of the manuscript. To select possible studies for inclusion in the definite analysis, the authors screened all available studies by title and, if suitable, by abstract. Duplicate manuscripts were excluded. Manuscripts that appeared relevant were read in full. All reference lists of studies that were read in full were screened for further readings. The whole review was conducted based on the Preferred Reporting Items for Systematic Reviews and Meta-Analyses (PRISMA) guidelines [10].

## 3. Results

**Figure 1:**
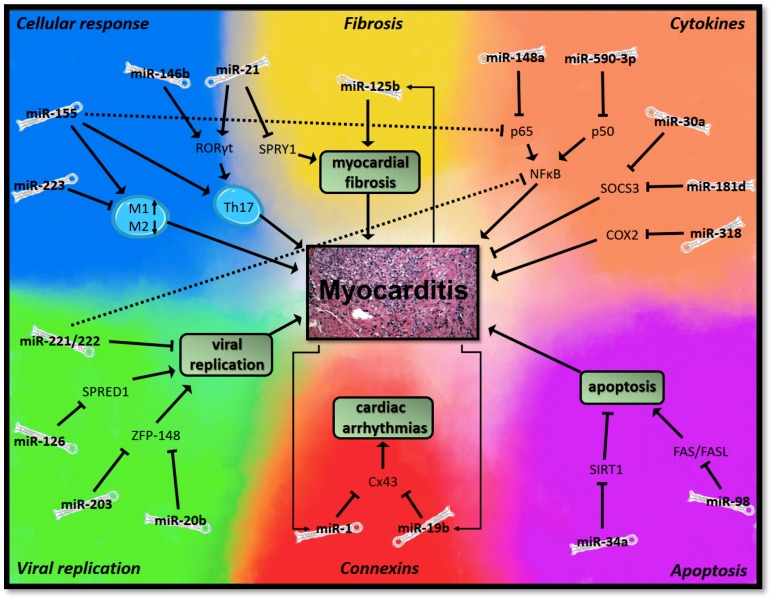
Schematic presentation of the putative mechanisms of the microRNAs (miRNAs) involved in myocarditis.

By searching the predefined search terms in the PubMed and Medline databases (search terms are listed in Appendix A of this manuscript), we identified a total of 131 studies, of which 96 abstracts were read. Of these, 69 original papers were read and included in the review (see Figure 1A in Appendix B).

Total Results: 125; Further readings: 6; Abstracts read: 96; Full-text read: 69; Included: 69.

### 3.1. MicroRNAs in Inflammatory Heart Diseases

#### 3.1.1. MicroRNAs in Myocarditis

Myocarditis is a common cause of sudden cardiac death (SCD) and dilated cardiomyopathy (DCM) [11,12]. In fact, current evidence suggests that up to 30% of patients with biopsy-proven myocarditis consequently develop DCM, which is associated with formidable symptoms and adverse outcomes [13,14]. Due to the lack of causative therapeutic approaches, the pathophysiologic processes behind myocarditis have been studied thoroughly within the last few decades. With the advent of miRNAs and the increasing understanding of their role in the pathophysiology of diseases, recent trials have focused on the microRNA transcriptome in animals and patients with myocarditis to elucidate possible therapeutic targets for the future. 

Interestingly, all of the investigated miRNAs seem to regulate immunological or pathophysiological processes by targeting similar pathways. In order to summarize the current evidence, without affecting the readability of this manuscript, we have subsumed these processes into six fields (see Figure 1).

##### Viral Replication

Current evidence suggests that several miRNAs facilitate viral replication in coxsackievirus B3 (CVB3)- induced myocarditis, thereby enhancing the progression of the disease. For example, miR-126 was found to target sprouty-related, EVH1-domain-containing protein 1 (SPRED1), thus inducing a feedback loop that leads to an increase in viral replication while also regulating the Wnt/β-catenin signaling pathway to promote virus-induced cell death and viral shedding [15]. Similarly, Tong et al. found that the passenger strand (*) of miR-10a significantly promotes viral biosynthesis in cell culture by directly targeting the 3D-coding region of the CVB3 genome, which encodes for the viral RNA polymerase [16] (before the annotation of the strands was changed to 5p/3p, the two strands were referred to as “guide strand” and “passenger strand” [17]). Other miRNAs were found to regulate apoptotic signals, thus generating a favorable environment for viral replication. For example, miR-20b and miR-203 were found to suppress the expression of zinc finger protein (ZFP)-148, which results in differential expression of prosurvival and proapoptotic genes, leading to enhanced cell survival that promotes further viral infection and replication [18,19]. Likewise, miR-590-5p was found to promote viral replication by blocking apoptotic signals by targeting sprouty homolog 1 (SPRY1) [20]. In contrast, Corsten et al. found that the miR-221/222 cluster acts protectively in CVB3-induced myocarditis via several targets influencing the immune response against viral replication [21].

##### Cellular Immune Response

By targeting the differentiation or polarization of immunological cells, various miRNAs modulate myocardial inflammation and hence influence the course of myocarditis. For example, Liu et al. found that miR-21 and miR-146b are upregulated in Balb/c mice with CVB3-induced myocarditis. Interestingly, silencing of either miR-21 or miR-146b resulted in a significantly reduced expression of the mRNA of RORγt, a critical factor of T-cell development, and consequently a decreased differentiation of Th17 leukocytes [22]. Besides miR-21 and miR-146b, current data suggests that miR-155 also promotes inflammation in viral myocarditis. In 2012, Corsten et al. found that silencing of mir-155 by LNA-anti-miR in a mouse model of CVB3-induced myocarditis resulted in decreased T-cell activation; reduced numbers of intracardiac monocyte-macrophages; and decreased levels of TNF-α, IL-6, IL-10, and IFN-γ [23]. More recently, Zhang et al. found that miR-155^−/−^ mice developed attenuated CVB3-induced myocarditis, expressed by less inflammatory infiltrates, decreased intracardiac CD45^+^ leukocytes, and decreased levels of IFN-γ. Moreover, silencing of miR-155 resulted in decreased differentiation of classically activated M1 macrophages and increased levels of M2 macrophages [24]. Furthermore, miR-155 was found to modulate the Th17/regulatory T cell (Treg) immune response, leading to an increased development of proinflammatory Th17 leukocytes and increasing their resistance to suppression by Treg-cells [25]. Hence, miR-155 could be a promising therapeutic target in the future. 

In contrast, Gou et al. found that miR-223 is downregulated in CVB3-induced myocarditis. In their study, miR-223 resulted in a suppression of markers attributable to the classically activated proinflammatory M1 macrophage phenotype (iNOS, TNF-α, and CD 86) and an increased expression of markers of the anti-inflammatory M2-phenotype (Arginase-1, Fizz-1, and CD 206) [26]. Therefore, miR-223 could act protectively in viral myocarditis by regulating macrophage polarization.

##### Cytokines

Several studies have investigated the role of miRNAs in the expression of cytokines by targeting transcription factors or enzymes in the setting of myocarditis. This subsection can only portray a selection of the manifold scientific papers that have been published on this matter [27,28].

NFκB is a key regulator of immunological processes in almost all living organisms [29], and several miRNAs were found to regulate myocardial inflammation by targeting it in myocarditis. For example, overexpression of miR-221 modulates the NFκB and the c-Jun N-terminal kinase (JNK) pathways, resulting in a suppression of the levels of IL-6 and TNF-α in H9c2 cells stimulated with LPS [30]. Similarly, mir-148a and miR-155 were found to suppress RelA (p65), a subunit of NFκB, thereby attenuating the expression of IL-6 and IL-1β [31]. Likewise, miR-590-3p was found to inhibit the p50 subunit of NFκB, thusly decreasing IL-6 and TNF-α expression in a model of experimental autoimmune myocarditis (EAM) in Lewis rats [32]. In contrast, miR-214 seems to promote myocardial inflammation by inhibiting ITCH, a suppressor of NFκB [33]. 

Since atherosclerosis constitutes a chronic inflammatory state of the vessel wall, NFκB is also involved in the pathophysiology of coronary artery disease. Interestingly, miR-181b was found to inhibit the NFκB-pathway in endothelial cells but not in leukocytes, which is why its relevance as an antiatherosclerotic target is currently subject to various investigative trials. Furthermore, the systemic delivery of miR-181b was found to decrease vascular inflammation in mice in a recent trial [34,35].

Besides NFκB, several other miRNAs regulate myocardial inflammation by targeting cytokine expression via transcription factors or enzymes. In a recent study, Pan et al. found that miR-141-3p is downregulated in Balb/c mice with EAM. Further experiments elucidated the role of miR-141-3p, which seems to suppress STAT4, a central component of the JAK/STAT pathway, which plays an important role in inflammatory processes [36]. Similarly, miR-318 is downregulated in patients with myocarditis, which results in an overexpression of cyclooxygenase 2 (COX2) that promotes inflammation [37]. In contrast, miR-30a and miR-181d were found to promote myocardial inflammation by targeting suppressor of cytokine signaling 3 (SOCS3), and an inhibition of these miRNAs significantly decreases mortality in a murine model of CVB3-induced viral myocarditis [38]. 

##### Apoptosis

Damage to the cardiomyocytes by the body’s immune response or the virus itself plays a significant role in the pathophysiology of myocarditis [39]. Several miRNAs were found to promote apoptotic processes in the setting of myocarditis, rendering them possible targets for novel therapeutic approaches. For example, Jiang et al. found that miR-34a was highly expressed in a cell culture model of CVB3-induced myocarditis and that it led to a downregulation of sirtuin 1 (SIRT1), which is the central component of the SIRT1–p53 signaling pathway and an important inhibitor of apoptosis [40]. In contrast, Zhang et al. found that miR-98 was downregulated in patients with myocarditis, whereas the expression of FAS/FASL was increased 1.68-fold. After performing experiments in cell culture, the authors found that miR-98 targets the FAS/FASL gene pair which can induce apoptosis [41].

##### Myocardial Fibrosis

Since myocardial fibrosis can be the result of chronic inflammation, it plays an important role in the progression of myocarditis to DCM. Recently, Xu et al. found that miR-21 is markedly upregulated in Balb/c mice with viral myocarditis and in patients with DCM. By targeting SPRY1 protein, miR-21 leads to an increased expression of the mitogen-activated protein kinase (MAPK), which results in myocardial fibrosis and cardiac remodeling [42]. Similarly, miR-125b was found to enhance fibrogenic pathways in mice with experimental autoimmune myocarditis because of an overexpression of the androgen receptor (AR) [43].

##### Connexins and Gap Junctions

Gap junctional channels couple adjacent cardiomyocytes, thereby establishing electrical conductance within the myocardium. The transmembrane proteins that constitute the building elements of these channels are called “connexins”, and recent evidence suggests that alterations of gap junction organization and connexin expression are associated with cardiac arrhythmias [44,45]. In viral myocarditis, miR-1 and miR-19b were found to negatively regulate the expression of connexin43 (Cx43), one of the most abundantly expressed connexins in heart tissue. Therefore, miR-1 and miR-19b might be involved in the pathogenesis of cardiac arrhythmias in viral myocarditis, which constitute a serious complication thereof [46,47,48].

#### 3.1.2. MicroRNAs in Endocarditis and Pericarditis

At the time of research for this review, the role of miRNAs in infective pericarditis and endocarditis has not yet been addressed in investigative studies. Interestingly, however, Ciccacci et al. identified a polymorphism of miR-1279 which promoted pericarditis in patients with systemic lupus erythematodes (SLE) [49], and Huang et al. found that miR-21 promotes myocardial fibrosis in a rat model of sterile pericarditis [50]. These results suggest that miRNAs play a role in the pathophysiology of pericarditis and therefore, further studies on this matter are certainly warranted.

### 3.2. MicroRNAs in Sepsis-Induced Cardiac Dysfunction

Sepsis represents a syndrome of physiologic, pathologic, and biochemical abnormalities caused by a systemic response to infection, which can result in dysfunctions of the lung, brain, liver, kidney, as well as the heart [4,51]. The underlying inflammatory processes are often accompanied by a hemodynamic impairment, which results in decreased tissue perfusion and cellular oxygen supply, thus aggravating end-organ damage [52,53]. Besides peripheral vascular dysfunction, sepsis-induced cardiac dysfunction is a key component of this macrocirculatory failure. Sepsis-induced cardiac dysfunction is present in half of all patients with septic shock [54] and is characterized by a deterioration of myocardial contractility, stroke volume, and diastolic compliance, thus leading to a significant impairment of left ventricular (LV) systolic and diastolic function [55]. Although the exact pathophysiologic processes are still not fully understood, current evidence suggests that alterations in cellular metabolism, adrenergic signaling, and calcium utilization could play a role in its pathogenesis. However, intramyocardial inflammation resulting from an activation of TLRs of cardiomyocytes, monocytes, and macrophages seems to be a key feature of sepsis-induced cardiac dysfunction [56,57].

Due to the manifold pathophysiologic processes involved, sepsis leads to a dysregulation of a wide variety of miRNAs, which is why miRNAs could aid in diagnosis and disease monitoring in this setting. In a recent trial by Zhang et al., the authors reported 78 differentially expressed miRNAs in the myocardium of rats with LPS-induced septic shock, among which miR-155-3p (2.8-fold increase) and miR-155-5p (1.9-fold increase) were upregulated the most and miR-20b-3p (-1.5-fold change) was downregulated the most [58]. Of these dysregulated miRNAs, several were associated with disease stage and adverse outcomes in previous trials [59]. For example, in a recent trial involving 93 patients (24 controls, 29 with sepsis, and 40 with septic shock), miR-150 could be associated with the extent of inflammation, severity of the disease, and mortality [60]. In another study involving 70 patients with sepsis and 30 patients with systemic inflammatory response syndrome (SIRS), decreased levels of miR-25 were associated with the severity of sepsis and adverse outcomes [61].

Since several miRNAs could be associated with adverse outcomes, the hypothesis arises that these might aggravate the inflammatory processes involved. In contrast, recent evidence suggests that other miRNAs might elicit protective effects in the setting of sepsis. Hence, the possibility of novel therapeutic approaches, such as the administration or inhibition of dysregulated miRNAs, has been subject to various investigative trials.

For example, the administration of miR-146a was found to reduce proinflammatory cytokines (TNF-α, IL-1β, and IL-1α) and inflammatory edema in an LPS-induced model of sepsis in rats by targeting the TLR-4/NFκB pathway [62]. This finding was confirmed in an in vitro model of LPS-stimulated H9c2 cells, where miR-146a improved cell viability and reduced apoptosis by suppressing IRAK1 and TRAF6, thereby negatively regulating the NFκB pathway [63]. Furthermore, miR-146a was found to improve sepsis-induced cardiac dysfunction, as assessed by ejection fraction (EF), fractional shortening (FS), and stroke volume (SV) in a rat model of cecal ligation and puncture (CLP) if administered prior to the induction of sepsis [64]. Besides in sepsis, miR-146a also elicits anti-inflammatory and atheroprotective effects on blood vessels by inhibiting the NFκB and MAPK signaling pathways in endothelial cells and macrophages [65,66].

Similar to miR-146a, two recent studies found that an administration of miR-125b attenuates inflammation and sepsis-induced cardiac dysfunction by targeting the NFκB pathway [67] and p53-mediated apoptotic signaling [68]. Moreover, miR-146b was recently found to negatively regulate inflammation in sepsis. Although significantly upregulated in LPS-induced sepsis, miR-146b seems to suppress the inflammatory processes involved, as portrayed by a reduction of IL-1β and cellular apoptosis, probably by targeting Notch1. Thereby, miR-146b possibly prevents an overwhelming inflammatory response in the setting of sepsis [69].

Conversely, Wang et al. recently found a significant downregulation of the miR-223 duplex (miR-223-3p and -5p) in a mouse model of CLP. The authors then generated knockout (KO) mice with a deletion of the miR-223 gene locus, which subsequently developed a remarkably aggravated inflammatory response to CLP, which resulted in a deterioration of cardiac function and survival. Hence, an administration of the miR-223 duplex could act protectively in the setting of sepsis [70]. 

In contrast, miR-21-3p was found to be upregulated in patients and mice with sepsis. Interestingly, an inhibition of miR-21-3p by antagomiR led to a preservation of cardiac function, as assessed by EF and FS, as well as improved survival in a murine model of LPS-induced sepsis [71]. Similarly, an inhibition of the upregulated miR-23b was found to result in an improvement of LV systolic function and cardiac fibrosis by suppressing TG-interacting factor 1 (TGIF1) and phosphatase and tensin homolog (PTEN), two negative regulators of fibrotic pathways [72].

In contrast to the aforementioned miRNAs, the role of miR-155, which is significantly upregulated in the context of sepsis, seems to be less clear. In fact, two recent trials have investigated miR-155 in animal models of sepsis and have found contradictory results. In a trial by Zhou et al., miR-155 was found to attenuate the course of CLP-induced sepsis by targeting JNK and Arrb2 [73], whereas a study by Wang et al. found that an inhibition of miR-155 by antagomiR results in preserved cardiac function and reduced cellular apoptosis in LPS-induced sepsis [74].

## 4. Discussion

When Lee et al. discovered microRNAs (miRNAs) in 1993 [75], they probably did not know what scientific interest they would set in motion. These small, single-stranded RNA sequences not only help us to improve our understanding of the physiological and pathophysiological processes at the cellular level but might become a valuable part of clinical practice in the future. 

From today’s perspective, dysregulated miRNAs could serve as biomarkers for diagnosis and disease monitoring in various disease entities, including inflammatory heart diseases and sepsis-induced cardiac dysfunction. Furthermore, considering the promising results of various animal models, miRNAs could become novel therapeutic targets, which could be substituted or inhibited by applying a synthetic “agomiR” or “antagomir”. However, further trials are warranted to investigate the utility of these experimental findings in clinical practice.

## Figures and Tables

**Figure A1:**
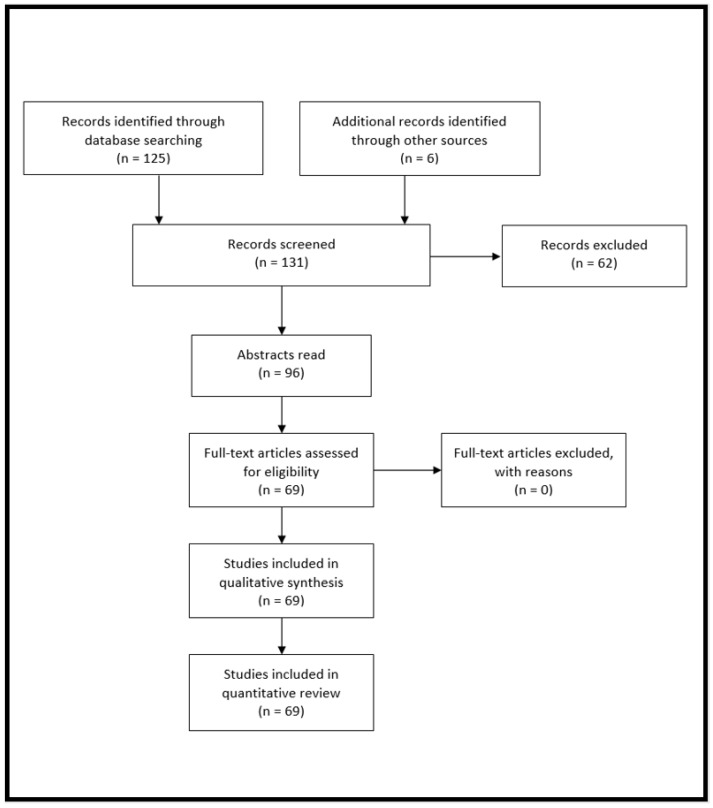
Flow diagram of the database search, screening, and inclusion of the studies (modified after the Preferred Reporting Items for Systematic Reviews and Meta-Analyses (PRISMA) guidelines [10]).

## Appendix A

### Appendix A.1. MicroRNAs in Inflammatory Heart Diseases

#### Appendix A.1.1. MicroRNAs in Myocarditis

Search Terms: “myocarditis + microRNA”, “myocarditis + miRNA”, “EAM + microRNA”, “EAM + miRNA”; Results: 92; Further readings: 2; Abstracts read: 59; Full-text read: 50; Included: 50.

#### Appendix A.1.2. MicroRNAs in Endocarditis and Pericarditis

Search Terms: “endocarditis + microRNA”, “endocarditis + miRNA”, “pericarditis + microRNA”, “pericarditis + miRNA”; Results: 4; Further readings: 0; Abstracts read: 4; Full-text read: 2; Included: 2.

### Appendix A.2. MicroRNAs in Sepsis-Induced Cardiac Dysfunction

Search Terms: “microrna + sepsis induced cardiac dysfunction”, “microrna + sepsis-induced cardiac dysfunction”, “mirna + sepsis induced cardiac dysfunction”, “mirna + sepsis-induced cardiac dysfunction”; Results: 29; Further readings: 4; Abstracts read: 33; Full-text read: 17; Included: 17.
